# Anesthetic Management of a Patient With Wolff-Parkinson-White Syndrome Undergoing Gynecological Robotic Surgery

**DOI:** 10.7759/cureus.62842

**Published:** 2024-06-21

**Authors:** Mohammed I Ismail, Omar R Al-Gharaibeh, Lana Talafha, Domenico Gammaldi, Giustino Varrassi, Giovanna Grasso

**Affiliations:** 1 Anesthesiology, Mut’ah School of Medicine, Alkarak, JOR; 2 Anesthesiology, Istishari Hospital, Amman, JOR; 3 Clinical Sciences, Faculty of Medicine, Yarmouk University, Irbid, JOR; 4 Anesthesiology, Casa di Cura Tortorella, Salerno, ITA; 5 Pain Medicine, Paolo Procacci Foundation, Rome, ITA; 6 Anesthesiology, Azienda Ospedaliera Universitaria (AOU) Federico II, Napoli, ITA

**Keywords:** myomectomy, eras, anesthesiology, robotic surgery, wpw syndrome

## Abstract

Robotic surgery provides precision and safety for minimally invasive gynecological operations but introduces unique anesthetic challenges, especially for individuals with pre-existing conditions like Wolff-Parkinson-White (WPW) syndrome. This case report addresses the anesthetic management of a 32-year-old female with WPW syndrome undergoing a myomectomy. A thorough pre-operative evaluation, including an ECG, echocardiogram, and Holter monitoring, was performed to assess the anesthetic and cardiac risks. The patient was administered a combination of loco-regional and general anesthesia, with an emphasis on neuromuscular monitoring, antiarrhythmic preparedness, and pain management to effectively manage the complexities introduced by WPW syndrome and robotic surgery. The anesthetic protocol comprised premedication with midazolam, induction using sufentanil, propofol, and rocuronium, and maintenance with desflurane, along with techniques to mitigate the effects of pneumoperitoneum and Trendelenburg positioning. Employing these strategies, the surgery concluded successfully without any anesthetic or surgical complications. The patient experienced a rapid and complete awakening, achieved optimal pain control, and was able to mobilize early, leading to her discharge 24 hours post-surgery. This case demonstrates the essential nature of customized anesthetic management for patients with WPW syndrome undergoing robotic surgery. It underscores the necessity of an exhaustive pre-operative assessment, diligent intraoperative monitoring, and active postoperative care to ensure patient safety and promote swift recovery.

## Introduction

Robotic surgery represents a new frontier of minimally invasive surgery and the evolution of laparoscopic surgery in terms of precision and safety, with a reduction of operating times, complications, and postoperative pain, and the use of opioids in the perioperative period; all this translates into shorter hospitalization times, so much so that robotic surgery can be fully included in the enhanced recovery after surgery (ERAS) protocols [1]. However, anesthetic management in robotic surgery represents a challenge for the anesthesiologist due to the changes that occur in the body's homeostasis [2,3]. These alterations occur mainly at the hemodynamic and respiratory levels due to the extreme position assumed by the patient (Trendelemburg 25-30°) and the pneumoperitoneum [2]. At the thoracic level, there is an increase in intrathoracic pressure and airway pressures, while from a hemodynamic point of view, the increase in intra-abdominal pressure resulting from the pneumoperitoneum associated with Trendelemburg leads to a reduction in venous return to the heart, a reduction in cardiac output, and the appearance of tachyarrhythmias or bradycardia, especially during the induction phase of the pneumoperitoneum [4-7]. These changes are more evident in patients with heart and/or respiratory diseases. Wolff-Parkinson-White syndrome (WPW) is a congenital electrophysiological disorder of nerve conduction of the heart due to the presence of abnormal electrical circuits between the atria and ventricles that result in ventricular pre-excitation [8]. Patients with this syndrome may be completely asymptomatic or have mild symptoms such as palpitations and heart palpitations, up to more severe symptoms such as dyspnea, syncope, and cardiac arrest [9]. On ECG, they have a short PR interval and prolonged QRS; sometimes there is an ST segment under elevation [10]. During anesthesia, serious perioperative complications such as atrial flutter (AF) or supraventricular tachycardia can occur, so it is important to maintain sinus rhythm and ensure an adequate anesthesia plan during all phases of the surgery to avoid the onset of cardiovascular complications [11]. Patients with WPW syndrome are susceptible to perioperative cardiac arrhythmias, which necessitates meticulous planning and management to mitigate risks during the anesthesia phase of robotic surgeries [12].

## Case presentation

We report the case of a 32-year-old patient with WPW syndrome who underwent a robotic myomectomy. During anesthesia counseling, the patient reports that she is not taking medication and is asymptomatic from a cardiovascular point of view, except occasionally for the appearance of palpitations. For the stratification of anesthetic and cardiac risk, the execution of the ECG, echocardiogram, 24-hour Holter ECG monitoring, and a pre-operative arrhythmological consultation were requested. On physical examination, blood pressure (BP) was 127/79 mmHg, and heart rate (HR) was 77 bpm. In the operating room, the patient received ECG, BP, saturation of peripheral oxygen (SpO2), and end-tidal carbon dioxide (EtCO2) monitoring, as well as neuromuscular monitoring with a train of four (TOF), and we prepared antiarrhythmic drugs and defibrillators for the management of any arrhythmias. After premedication with midazolam 0.07 mg/kg, we performed a combination of loco-regional and general anesthesia. A subarachnoid T8-T9 anesthesia was performed for analgesic purposes with local anesthetics (ropivacaine 0.375 7.5 mg) and adjuvants (dexmedetomidine 7 mcg) for pain control in the perioperative period to avoid the establishment of a sympathetic nervous system response to stress and pain. Afterward, we performed general anesthesia; after pre-oxygenation of the patient with 100% O2 at induction, we administered sufentanil (5 mcg), propofol (1.5 mg/kg), and rocuronium bromide (0.6 mg/kg), then we performed the laryngoscopy maneuver for intubation orotracheal or orotracheal intubation (IOT). Maintenance of anesthesia was performed with desflurane 5-6% by titrating the concentration to obtain a minimal alveolar concentration (MAC) of 1. The patient received gastric prophylaxis (pantoprazole 20 mg) during surgery. Induction of pneumoperitoneum was achieved gradually and by open laparoscopy (Hasson's technique) to avoid the high intra-peritoneal pressure that was kept constant throughout the surgery and not exceeding 12 mmHg. EtCO2 was maintained between 32 and 36 mmHg, with airway pressures not exceeding 40 mmHg. We found a slight increase in blood pressure (BP) and cardiac function (CF) only during the induction of pneumoperitoneum and the subsequent establishment of the Trendelemburg position, which stabilized shortly after a deepening of the anesthesia plan by increasing the concentration of desflurane. The surgery lasted about 90 minutes without any anesthetic or surgical complications. Before the patient was awakened, surgical access sites were infiltrated with ropivacaine. The patient received prophylaxis for postoperative nausea and vomiting (PONV) for 30 minutes before awakening (dexamethasone 4 mg + ondansetron 4 mg iv) and paracetamol 1000 mg iv. In the awakening phase, neuromuscular blockade was antagonized with sugammadex 3 mg/kg under TOF guidance to proceed with tracheal extubation safely and avoid postoperative residual curarization (PORC) syndrome. Awakening was rapid and complete in the operating room in the absence of pain (numerical rating scale (NRS) 0), postoperative nausea and vomiting, and mobility of the lower limbs (Aldrete score 10/10). The patient was monitored in the recovery room for 30 minutes after the surgery and then transferred to the ward. In the inpatient ward, the patient was mobilized early, properly hydrated, and resumed oral fluid intake a few hours after surgery to minimize postoperative fasting times as required by enhanced recovery after surgery (ERAS) protocols. Paracetamol was prescribed every eight hours after surgery for the first 24 hours. The patient only needed one rescue dose of ketorolac (30 mg) on the evening of surgery. The next day, 24 hours after the surgery, she was discharged home.

## Discussion

This case report outlines the comprehensive anesthetic management of a 32-year-old patient with Wolff-Parkinson-White (WPW) syndrome undergoing myomectomy via robotic surgery. The approach demonstrates a meticulous preparation and execution strategy tailored to the patient's unique cardiac condition and surgical demands.

Pre-operative evaluation

The patient's pre-operative assessment included a thorough cardiovascular evaluation with an ECG, echocardiogram, 24-hour Holter monitoring, and arrhythmological consultation, highlighting the importance of a detailed cardiac risk stratification in patients with WPW syndrome. The physical examination and baseline vitals were within normal limits, providing a stable starting point for anesthesia management. The literature emphasizes the importance of meticulous planning for patients with WPW syndrome undergoing surgery, as they are at increased risk for perioperative arrhythmias [13]. This case's strategy, including the preparedness with antiarrhythmic drugs and defibrillators, aligns with recommended practices for managing potential arrhythmic events in such patients.

Anesthetic management

The anesthetic strategy involved a combination of loco-regional and general anesthesia to optimize pain control while minimizing the sympathetic nervous system response. This dual approach underscores the significance of maintaining hemodynamic stability and pain management in patients with cardiac anomalies, particularly those with WPW syndrome. The use of midazolam for premedication, followed by a tailored regimen of sufentanil, propofol, and rocuronium for induction, and desflurane for maintenance, reflects a balanced and patient-specific anesthetic plan. Studies have demonstrated that combining loco-regional anesthesia with general anesthesia can enhance postoperative pain control, reduce opioid consumption, and potentially decrease the risk of postoperative complications [14].

Intraoperative monitoring and management

The detailed monitoring setup, including ECG, BP, SpO2, EtCO2, and neuromuscular monitoring with TOF, alongside the readiness of antiarrhythmic drugs and defibrillators, emphasizes the preparedness for managing potential arrhythmias. The careful induction of pneumoperitoneum using Hasson's technique to avoid high intra-peritoneal pressure and the meticulous management of EtCO2 and airway pressures demonstrate a keen awareness of the physiologic changes in robotic surgery and their impact on patients with WPW syndrome. Research indicates that the induction of pneumoperitoneum and the use of the Trendelenburg position can have significant hemodynamic and respiratory effects, particularly in patients with pre-existing cardiac conditions [15].

Postoperative care and recovery

Postoperative care, including PONV prophylaxis, pain management with paracetamol and rescue toradol, and early mobilization, aligns with ERAS protocols, underscoring the role of anesthesiology in enhancing recovery [16]. The successful management of neuromuscular blockade reversal with sugammadex and the achievement of rapid, complete awakening with optimal recovery scores highlight the effectiveness of the anesthetic technique in ensuring patient safety and comfort [17].

## Conclusions

This case report exemplifies the critical role of tailored anesthetic management in robotic surgery for patients with WPW syndrome. It demonstrates that with careful pre-operative planning, vigilant intraoperative monitoring, and proactive postoperative care, patients with complex cardiac conditions can safely undergo major robotic surgeries, achieving excellent surgical and anesthetic outcomes. The report contributes valuable insights into the anesthetic considerations and strategies essential for optimizing care in this unique patient population.
